# Association of the *PINX1* Variant rs6984094, Which Lengthens Telomeres, with Systemic Lupus Erythematosus Susceptibility in Chinese Populations

**DOI:** 10.1155/2021/7079359

**Published:** 2021-07-13

**Authors:** Yuan-yuan Qi, Xin-ran Liu, Ying-xin He, Min Zhou, Xiang-hui Ning, Ya-ling Zhai, Xiao-xue Zhang, Xiao-yang Wang, Ya-fei Zhao, Yan Cui, Zhan-Zheng Zhao

**Affiliations:** ^1^Nephrology Hospital, The First Affiliated Hospital of Zhengzhou University, Zhengzhou University, Henan 4500052, China; ^2^School of Pharmaceutical Sciences, Zhengzhou University, 100 Ke Xue Avenue, Zhengzhou, Henan 450001, China; ^3^Department of Urology, The First Affiliated Hospital of Zhengzhou University, Henan 4500052, China

## Abstract

A recent genome-wide association study (GWAS) of Asian ancestry reported that single nucleotide polymorphism (SNP) in *TERT* (telomerase reverse transcriptase) was associated with systemic lupus erythematosus (SLE). TERT has a critical role in maintaining the chromosomal stability and the length of telomere. Given that only a small portion of the genetic heritability of SLE has been explained so far, we aimed to identify novel loci in telomere-related genes responsible for SLE susceptibility in Chinese populations. We performed a comprehensive genetic association analysis of SLE with telomere-related genes. To identify functional significance, we analyzed the publicly available HaploReg v4.1 and RegulomeDB databases. Differential gene expression analysis was also performed using ArrayExpress. A novel signal of *PINX1* rs6984094 was identified (*P*_discovery_ = 4.13 × 10^−2^, OR = 0.58, 95% CI 0.35-0.98) and successfully replicated (*P*_replication_ = 5.73 × 10^−3^, OR = 0.45, 95% CI 0.26-0.81). Multiple layers of functional analysis suggested that the *PINX1* rs6984094 risk T allele exhibited increased nuclear protein binding. We also observed an increased expression of *PINX1* mRNA in peripheral blood mononuclear cells from SLE patients compared with healthy controls. Overall, we observed a novel genetic association between *PINX1* (encodes the PinX1 protein, an inhibitory telomerase enzyme that lengthens telomeres) and SLE susceptibility in Chinese populations.

## 1. Introduction

Systemic lupus erythematosus (SLE [MIM152700]), a prototypical autoimmune disease with diverse clinical manifestations, has no clear etiology and is a challenge to clinicians [1]. Compelling evidence suggests that genetic factors contribute to the pathogenesis of SLE. In the past few years, genome-wide association studies (GWAS) have identified more than 80 susceptibility loci which achieved great progress [2]. However, the genetic background for SLE remains to be elucidated, as the known genetic risk is less than 30% [2]. Thus, further exploration of GWAS data is required to improve our understanding of the genetic background of SLE.

Telomeres, specialized nucleoprotein structures, protect the natural ends of linear chromosomes to maintain chromosome stability. Telomerase, a specific telomere-terminal transferase, counteracts telomere shortening after each cellular division. Telomeric erosion induces cell apoptosis, and the clearance impairment of apoptotic cells is the hallmark of SLE [3]. Accelerated telomere shortening has been observed in SLE patients [4–7] regardless of ethnicity, sample type, or assay method [7]. Telomere shortening of polymorphonuclear neutrophils in SLE patients is related to SLE disease activity (as measured by the Systemic Lupus Erythematosus Disease Activity Index (SLEDAI)) [4]. A longer telomere length is associated with steroid therapy [6]. The telomerase activity of peripheral blood mononuclear cells (PBMCs) has been identified to be significantly increased in SLE patients [8–10] and correlated with the modified SLEDAI [8]. Abnormalities in telomerase activity and telomere length have been observed in SLE patients which has expanded our understanding of the mechanistic underpinnings of SLE pathogenesis.

A recent GWAS of Asian ancestry including Korean, Beijing Han Chinese, Shanghai Han Chinese, and Japanese populations reported that *TERT* rs7726159 was associated with SLE [11]. Telomerase reverse transcriptase (TERT) is critical to the maintenance of chromosomal stability and the length of telomeres. Given the potential role of telomere or telomerase genes in the pathogenesis of SLE, it is crucial to investigate the genetic contributions of such genes in SLE.

In this study, we performed a comprehensive genetic association analysis of genes known to be related to telomeres or telomerases based on the HUGO Gene Nomenclature Committee (HGNC) database. The discovery Beijing cohort (490 SLE patients vs. 493 healthy donors) was adopted from previous GWAS by Sun et al. [11]. We recruited 1003 SLE patients and 815 healthy donors from the Middle East of China as the independent replication cohort. *TERT* rs7726159 was successfully replicated in our replication cohort, and a new signal was identified. Moreover, we integrated multiple annotation databases including HaploReg, RegulomeDB, and ArrayExpress to prioritize the plausible function of the new signal.

## 2. Method

### 2.1. Case-Control Subjects

The discovery cohort consisted of 490 SLE patients and 493 healthy controls of Chinese Han Beijing which were adopted from previously reported GWAS data [11]. Detailed descriptions of the samples, data quality, and genomic controls can be found in the original GWAS [11]. For replication analysis, we recruited 1003 sporadic SLE patients and 815 geographically matched unrelated healthy controls of the Henan population from the Middle East of China. Clinical manifestations of SLE patients in the replication cohort are presented in Supplementary Table 1. All the patients fulfilled the criteria of the American College of Rheumatology for SLE. The study was approved by the Medical Ethics Committee of Zhengzhou University First Hospital.

### 2.2. SNP Selection and Genotyping

A total of 21 telomere-related genes were identified by searching “telomere” (9 genes: *CTC1*, *SDE2*, *TELO2*, *RTEL1*, *RTEL1P1*, *TERB1*, *TERB2*, *ATM*, and *HMBOX1*) and “telomerase” (12 genes: *PINX1*, *ACD*, *TEP1*, *TERC*, *TERT*, *TRIR*, *TEN1*, *SCYL1*, *SMG5*, *SMG6*, *SMG7*, and *WRAP53*) in the HGNC database (http://www.genenames.org). Twelve genes (*SDE2*, *TELO2*, *RTEL1P1*, *TERB1*, *TERB2*, *ATM*, *HMBOX1*, *TERC*, *TRIR*, *SCYL1*, *SMG5*, and *WRAP53*) were not included by ImmunoChip [11]. Therefore, the SNPs in the remaining 9 genes (*CTC1*, *RTEL1*, *PINX1*, *ACD*, *TEP1*, *TERT*, *TEN1*, *SMG6*, and *SMG7*), along with those 10 kb upstream and 10 kb downstream, were enrolled for further genetic analysis as the discovery cohort [11]. We extracted the results of all available SNPs from previously reported GWAS. A total of 458 SNPs were covered and 6 SNPs failed to be genotyped successfully by ImmunoChip [11]. For genotyping in the replication cohort, we used Sequenom MassARRAY, and the genotyping yield was 96~100%.

### 2.3. Bioinformatic Analysis and Functional Prediction

The regulatory elements of the variants were annotated by searching the HaploReg v4.1 (http://archive.broadinstitute.org/mammals/haploreg) and RegulomeDB (http://regulome.stanford.edu) databases. Global distributions of rs6984094 among 53 populations were derived from the HGDP Selection Browser (http://hgdp.uchicago.edu/) [12–14]. Differential gene expression analysis of *PINX1* was performed in PBMCs (E-GEOD-50772) using ArrayExpress (http://www.ebi.ac.uk/arrayexpress) [15].

### 2.4. Statistical Analysis

Differences in the frequencies of genotypes between the patients and controls were tested by the chi-squared test. Odds ratios and corresponding 95% confidence intervals were calculated to determine the relative SLE risk. Analyses were performed by using SPSS 19.0 software. A two-sided *P* value < 0.05 was set as the statistical significance level.

## 3. Results

### 3.1. SNPs of Telomere-Related Genes Associated with SLE Susceptibility

In the discovery stage, 6 SNPs from 5 telomere-related gene regions (*RTEL1*, *RTEL1*-*TNFRSF6B*, *PINX1*, *TERT*, and *BICD1*) were associated with SLE susceptibility (*P* < 0.05).

In the replication stage, rs7726159 on *TERT*, which was identified to be associated with SLE susceptibility in a previously reported GWAS, was also included for further replication study [11]. The association of *TERT* rs7726159, identified by the previous GWAS, with susceptibility to SLE in Asians, including those of Korean, Beijing Han Chinese, Shanghai Han Chinese, and Japanese descent, was also confirmed (*P* = 3.47 × 10^−3^, OR = 1.22, 95% CI 1.07-1.39) by an independent cohort from a Henan Han population of China.

More importantly, a novel signal of *PINX1* rs6984094 was identified (*P*_discovery_ = 4.13 × 10^−2^, OR = 0.58, 95% CI 0.35-0.98) and successfully replicated (*P*_replication_ = 5.73 × 10^−3^, OR = 0.45, 95% CI 0.26-0.81) (Table 1).

### 3.2. Regulatory Effects of *PINX1* rs6984094 Predicted by Bioinformatic Analysis

**Figure 1:**
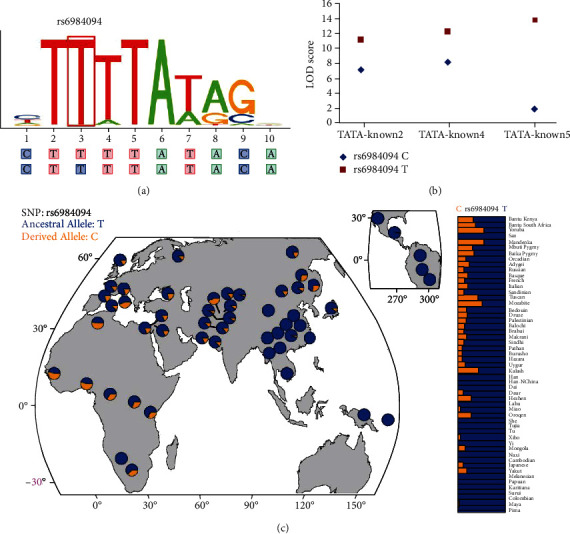
Bioinformatic analysis supports the functional role of systemic lupus erythematosus-associated rs6984094. (a) According to the RegulomeDB database, the relative height of each letter is proportional to its overenrichment in the motif. A line is boxed around rs6984094-T; this systemic lupus erythematosus-associated allele is predicted to form the 3rd nucleotide in the motif. (b) Altering the rs6984094 allele from protective allele C to risk allele T increased the binding affinity for transcription factors TATA_Known2, TATA_Known4, and TATA_Known5 according to HaploReg v4.1 database. (c) Genetic signatures of population selection in the human genome. Shown is the detailed global allele frequency distribution of the single-nucleotide polymorphism (SNP) rs6984094 in 53 world populations.

Since rs6984094 is located in the intronic region, we conducted bioinformatic analysis using ENCODE data. Notably, the RegulomeDB score of rs6984094 reached 6, indicating that it contains predicted transcription factor binding motifs and is located in the histone modification regions (Supplementary Table 2). Both the RegulomeDB and HaploReg databases indicated that the TATA binding site motif spanned the rs6984094 region (Figures 1(a) and 1(b)). The differences between the LOD scores for alleles C and T (reference) were -4, -4.2, and -12 for TATA_known2, TATA_known4, and TATA_known5, respectively. Therefore, this model showed a higher binding affinity for the risk T allele than the protective C allele (Figure 1(b)).

In addition, 47 proxy SNPs were closely linked (*r*^2^ > 0.8) with rs6984094 (Supplementary Table 3). All of the 47 SNPs showed regulatory effects according to the HaploReg database: 5 SNPs were located within the regions of promoter histone marks, 28 within the regions of enhancer histone marks, 18 within the regions of DNase-I hypersensitivity, 6 within the regions of protein binding, 45 in motif regions, and 8 within expression quantitative trait loci (eQTLs) (Supplementary Table 3).

Collectively, our functional predictions identified that rs6984094 might have potential functional roles in regulating *PINX1* expression, which need to be tested in the future.

### 3.3. Population Selection Analysis of *PINX1* rs6984094

The global allele frequency distribution of rs933717 in 53 populations is shown in Figure 1(c). Interestingly, the risk allele rs6984094T showed regional enrichment: the highest frequencies (~100%) were found in Asia, followed by the Middle East and Europe, and the lowest were found in Africa.

### 3.4. Higher Expression of *PINX1* in SLE Patients

**Figure 2:**
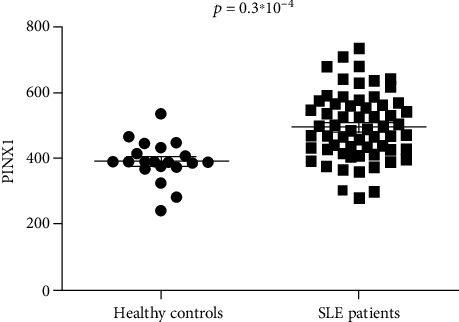
Increased levels of mRNA expression of *PINX1* in systemic lupus erythematosus. The levels of *PINX1* mRNA expression in the peripheral blood mononuclear cells from 61 SLE patients and 20 healthy controls (E-GEOD-50772) were presented.

Given the genetic association analysis and functional annotation of the rare variant rs6984094 in *PINX1*, we speculated that *PINX1* might play a role in SLE. Furthermore, we explored the expression levels of *PINX1* in SLE patients using the ArrayExpress database. As shown in Figure 2, the levels of *PINX1* mRNA expression were significantly upregulated in SLE patients compared with healthy controls (median, interquartile range of normalized fluorescence intensity: 20 healthy controls 389.7 374.0-427.3 vs. 61 SLE patients 484.6 426.4-566.0; *P* = 0.3∗10^−4^).

## 4. Discussion

Herein, we investigated the genetic association between telomere-related gene polymorphisms and SLE susceptibility in a Chinese population. In this study, a significant association between *PINX1* rs6984094 and SLE susceptibility was identified. The risk T allele showed a higher affinity for transcription factors that might affect the transcription of *PINX1*. Accordingly, a higher level of *PINX1* mRNA expression was observed in SLE patients than in healthy donors. However, no significant correlation was observed between *PINX1* rs6984094 genotypes and clinical manifestations of SLE patients due to the low frequency of minor alleles (Supplementary Table 4).

With the constant encounter of immune stimulation, T and B lymphocytes remain in a long-term proliferation state [16, 17]. Every time a cell divides, its telomeres are shortened promoting genome instability and cell apoptosis below a certain threshold. Thus, telomeric shortening rendering T and B lymphocytes susceptible to apoptosis is a potential underlying risk factor causing autoimmune diseases including SLE [16, 17]. In the present study, we revealed that *PINX1* rs6984094 risk T allele exhibited an increased nuclear protein binding, and an increased expression of *PINX1* mRNA was also observed in PBMC from SLE patients. The PinX1 protein, encoded by *PINX1*, inhibits telomerase, an enzyme that lengthens telomeres. Loss of PinX1 leads to increased telomere length contributing to the development of cancer [18]. In contrast, overexpression of *PINX1* suppresses telomerase activity, resulting in telomere shortening [19]. Given our findings, we hypothesized that individuals carrying PINX1 rs6984094 risk T allele might have a higher level of *PINX1* expression by an increased nuclear protein binding compared with those carrying protective T allele. By suppressing telomerase activity, the augmented *PINX1* expression accelerated telomere shortening which had already been reported in several SLE cohorts.

*PINX1* has been reported to be associated with systemic sclerosis (SSc (scleroderma)) in both African-American and White populations [20]. SSc is an autoimmune disease featured by fibrosis, vasculopathy, and autoantibody production. Ectopic overexpression of *PINX1* in CRC cells has been reported to inhibit cell proliferation, promote apoptosis, repress telomerase activity, and induce telomere shortening [21]. Additionally, *PINX1* overexpression significantly inhibits telomerase activity, inhibits cell growth, arrests cells at the G0/G1 stage, and induces cell apoptosis in Eca109 cells [22]. Given that telomeric erosion induces cell apoptosis and that the clearance impairment of apoptotic cells is the hallmark of SLE pathogenesis [3], the increased expression of *PINX1* might contribute to telomere shortening and cell apoptosis in patients with SLE.

Our present study also confirmed previously reported locus *TERT* rs7726159 in the replication cohort. According to the GWAS data, the significance of *TERT* rs7726159 and SLE susceptibility increased with an enlarged sample size [11]. With a sample size of approximately 1000 individuals, the *P* value ranged from marginal significance 0.161 (Beijing Han Chinese cohort in the discovery stage with 500 SLE patients and 500 healthy donors) to 0.012 (Shanghai Han Chinese cohort in the replication stage with 501 SLE patients and 622 healthy donors). The significance of the genetic associations in the Japanese GWAS dataset (891 cases and 3384 controls in the replication stage) and South Korean dataset (1843 SLE patients and 3262 controls in the discovery stage) was enhanced with *P* values of 7.60∗10^−3^ and 2.10∗10^−4^, respectively. In our present study, the sample size was approximately 1000 (314 SLE patients and 815 healthy donors), and the level of genetic association (*P* value 0.02) was comparable with previous GWASs, which improved the reliability of our genetic association result. *TERT* rs7726159 is also a functional variant with enhanced binding affinity with risk rs7726159 A allele compared with protective C allele according to HaploReg v4.1. However, the underlying mechanisms of risk rs7726159 A allele in promoting the pathogenesis of SLE warrants further investigation.

Several limitations exist in our study. First, although we included all the SNPs in telomere-related genes detected by ImmunoChip, 12 genes were covered, and we may miss some key variants. Future studies are necessary to explore the genetic associations within those genes and SLE susceptibility. Second, the discovery cohort and replication cohort were recruited from the North and Middle East of China (the Beijing population and Henan population, respectively), which inevitably might cause selection bias applying to the general population in China. Validations from more centers and larger sample sizes are crucial to get a solid genetic association result. Additionally, in vivo or in vitro studies are warranted to reveal the underlying mechanisms of potentially functional PINX1 rs6984094.

In conclusion, our study provided evidence that telomere-related gene *PINX1* rs6984094 polymorphisms may play a role in the pathogenesis of SLE. Further replication studies with larger sample sizes and functional experiments are warranted to confirm our findings.

## Figures and Tables

**Table 1:** Association analysis of single nucleotide polymorphisms from telomere-related genes with susceptibility to systemic lupus erythematosus in Chinese populations.

Chr.	Gene	SNP	Position (hg19)	Minor allele	Discovery stage (490/493)			Replication stage (1003/815)		
					MAF (case/control %)	*P* value	OR (95% CI)	MAF (case/control %)	*P* value	OR (95% CI)
20	*RTEL1*	rs2738783	62308612	T	45.2/40.7	4.23∗10^−2^	1.2 (1.01-1.44)	43.0/41.5	0.366	1.06 (0.93-1.21)
20	*RTEL1-TNFRSF6B*	rs6062496	62329099	A	11.2/15.1	1.03∗10^−2^	0.71 (0.54-0.92)	13.0/13.0	0.999	1.00 (0.82-1.22)
8	*PINX1*	rs10089869	10634324	T	3.4/5.3	3.77∗10^−2^	0.63 (0.40-0.98)	2.8/2.8	0.944	1.01 (0.68-1.51)
8	*PINX1*	rs6984094	10645738	C	2.3/4	4.13∗10^−2^	0.58 (0.35-0.98)	1.0/2.1	5.73∗10^−3^	0.46 (0.26-0.81)
5	*TERT*	rs2853676	1288547	A	19/14.1	3.57∗10^−3^	1.43 (1.12-1.81)	20.0/18.1	0.154	1.13 (0.96-1.34)
		rs7726159	1282319	A	44/42	1.61∗10^−1^	1.09 (0.91-1.31)	46.6/41.7	3.41∗10^−3^	1.22 (1.07-1.39)
12	*BICD1*	rs1798613	32277522	T	23.2/19.5	4.57∗10^−2^	1.25 (1.00-1.55)	22.9/22.7	0.883	1.01 (0.87-1.18)

## Data Availability

The data that support the findings of this study are available from the corresponding author upon reasonable request.

## Abbreviations

SLE: Systemic lupus erythematosus
GWAS: Genome-wide association study
SLEDAI: SLE disease activity
PBMC: Peripheral blood mononuclear cells
eQTL: Expression quantitative trait loci
SNP: Single nucleotide polymorphism
TERT: Telomerase reverse transcriptase
PINX1: TERF1-interacting telomerase inhibitor 1.

## Supplementary Materials

Supplementary Materials Supplementary Table 1: clinical manifestations of SLE patients in the replication cohort. Supplementary Table 2: functional annotations of rs6984094 by RegulomeDB. Supplementary Table 3: functional annotations of rs6984094 and variants with *r*^2^ ≥ 0.8 by HaploReg v4.1. Supplementary Table 4: the associations between genotypes of rs6984094 and rs7726159 and clinical manifestations of SLE patients in the replication cohort.
