# Supplementary material for: Association of the PINX1 Variant rs6984094, Which Lengthens Telomeres, with Systemic Lupus Erythematosus Susceptibility in Chinese Populations
Source: J Immunol Res. 2021 Jul 13;2021:7079359. doi: 10.1155/2021/7079359 (PMC8294968; doi:10.1155/2021/7079359)
Supplement: Supplementary Materials — Supplementary Table 1: clinical manifestations of SLE patients in the replication cohort. Supplementary Table 2: functional annotations of rs6984094 by RegulomeDB. Supplementary Table 3: functional annotations of rs6984094 and variants with r2 ≥ 0.8 by HaploReg v4.1. Supplementary Table 4: the associations between genotypes of rs6984094 and rs7726159 and clinical manifestations of SLE patients in the replication cohort. [file 7079359.f1.docx]

**Association of the *PINX1* Variant rs6984094*,* which Lengthens Telomeres, with Systemic Lupus Erythematosus Susceptibility in Chinese Populations**

Yuan-yuan Qi^1#^, Xin-ran Liu^1#^, Ying-xin He^3^, Min Zhou^3^, Xiang-hui Ning^2^, Ya-ling Zhai^1^, Xiao-xue Zhang^1^, Xiao-yang Wang^1^, Ya-fei Zhao^1^, Yan Cui^1^, Zhan-Zheng Zhao^1*^

**AUTHORS’ INSTITUTION AND AFFILIATION**

1. Nephrology Hospital, the First Affiliated Hospital of Zhengzhou University, Zhengzhou University, Henan 4500052, China;
2. Department of Urology, the First Affiliated Hospital of Zhengzhou University, Henan 4500052, China;
3. School of Pharmaceutical Sciences, Zhengzhou University, 100 Ke xue Avenue, Zhengzhou, Henan 450001, China.

^#^: These authors contribute equally to this work.

CORRESPONDING AUTHOR

Dr.Zhan-zheng Zhao, MD & PhD;

Email: zhanzhengzhao@zzu.edu.cn

Nephrology Hospital, the First Affiliated Hospital of Zhengzhou University,

Institute of Nephrology, Zhengzhou University

No.1, Jianshe Road, Erqi District

Zhengzhou 4500052, P.R China

Concise description:

Supplementary Table 1. Clinical Manifestations of SLE patients in the replication cohort.

Supplementary Table 2. Functional annotations of rs6984094 by RegulomeDB.

Supplementary Table 3. Functional annotations of rs6984094 and variants with r^2^ >= 0.8 by HaploReg v4.1.

Supplementary Table 4. The associations between genotypes of rs6984094 and rs7726159, and clinical manifestations of SLE patients in the replication cohort.Supplementary table 1. Clinical manifestations of SLE patients in the replication cohort.

| Clinical Manifestations | SLE (n = 1003) |
| --- | --- |
| Age onset (year, mean±SD) | 31.3±12.9 |
| Sex (female, %) | 932 (92.9) |
| Malar rash (+, %) | 253 (25.2) |
| Discoid rash (+, %) | 7 (0.7) |
| Photosensitivity (+, %) | 42 (4.2) |
| Oral ulcers (+, %) | 73 (7.3) |
| Arthritis (+, %) | 279 (27.8) |
| Serositis (+, %) | 83 (8.3) |
| Renal disorder (+, %) | 509 (50.9) |
| Neurological disorder (+, %) | 36 (3.6) |
| Hematological disorder (+, %) | 543 (55.6) |
| Anti-dsDNA antibodies (+, %) | 571 (63.0) |
| SLEDAI (mean±SD) | 4.7±4.1 |

Supplementary Table 2. Functional annotations of rs6984094 by RegulomeDB.

| Method | Location | Motif | [Cell Type](http://genome.ucsc.edu/ENCODE/cellTypes.html) | PWM | Reference |
| --- | --- | --- | --- | --- | --- |
| PWM | chr8:10645735..10645745 | TATA |  |  | [16381825](http://www.ncbi.nlm.nih.gov/pubmed/16381825) |
| B. Histone modifications | |  |  |  |  |
| Method | Location | Chromatin State | Tissue Group | Tissue | Reference |
| ChromHMM | chr8:10594800..10668400 | Quiescent/Low | Digestive | Colonic Mucosa | [REMC](http://www.roadmapepigenomics.org/) |
| ChromHMM | chr8:10597200..10646800 | Quiescent/Low | Other | Fetal Kidney | [REMC](http://www.roadmapepigenomics.org/) |
| ChromHMM | chr8:10610400..10661800 | Quiescent/Low | Blood & T-cell | Primary T helper memory cells from peripheral blood 2 | [REMC](http://www.roadmapepigenomics.org/) |
| ChromHMM | chr8:10610800..10662000 | Weak transcription | Blood & T-cell | Primary T CD8+ naive cells from peripheral blood | [REMC](http://www.roadmapepigenomics.org/) |
| ChromHMM | chr8:10611000..10657000 | Quiescent/Low | Digestive | Rectal Mucosa Donor 31 | [REMC](http://www.roadmapepigenomics.org/) |
| ChromHMM | chr8:10618800..10646200 | Weak transcription | Epithelial | Foreskin Fibroblast Primary Cells skin02 | [REMC](http://www.roadmapepigenomics.org/) |
| ChromHMM | chr8:10618800..10665200 | Weak transcription | Brain | Fetal Brain Female | [REMC](http://www.roadmapepigenomics.org/) |
| ChromHMM | chr8:10619200..10656400 | Weak transcription | Sm. Muscle | Rectal Smooth Muscle | [REMC](http://www.roadmapepigenomics.org/) |
| ChromHMM | chr8:10619400..10661600 | Weak transcription | Blood & T-cell | Primary T cells effector/memory enriched from peripheral blood | [REMC](http://www.roadmapepigenomics.org/) |
| ChromHMM | chr8:10620000..10669800 | Weak transcription | Digestive | Rectal Mucosa Donor 29 | [REMC](http://www.roadmapepigenomics.org/) |
| ChromHMM | chr8:10621200..10662000 | Weak transcription | Blood & T-cell | Primary T helper naive cells from peripheral blood | [REMC](http://www.roadmapepigenomics.org/) |
| ChromHMM | chr8:10623200..10650000 | Weak transcription | Digestive | Duodenum Mucosa | [REMC](http://www.roadmapepigenomics.org/) |
| ChromHMM | chr8:10623400..10646400 | Weak transcription | Epithelial | Foreskin Fibroblast Primary Cells skin01 | [REMC](http://www.roadmapepigenomics.org/) |
| ChromHMM | chr8:10623400..10656800 | Weak transcription | Sm. Muscle | Duodenum Smooth Muscle | [REMC](http://www.roadmapepigenomics.org/) |
| ChromHMM | chr8:10623400..10662600 | Weak transcription | Thymus | Fetal Thymus | [REMC](http://www.roadmapepigenomics.org/) |
| ChromHMM | chr8:10623400..10662800 | Weak transcription | HSC & B-cell | Primary monocytes from peripheral blood | [REMC](http://www.roadmapepigenomics.org/) |
| ChromHMM | chr8:10623400..10665000 | Weak transcription | Digestive | Fetal Intestine Large | [REMC](http://www.roadmapepigenomics.org/) |
| ChromHMM | chr8:10623600..10648800 | Weak transcription | Digestive | Fetal Intestine Small | [REMC](http://www.roadmapepigenomics.org/) |
| ChromHMM | chr8:10623800..10648800 | Weak transcription | HSC & B-cell | Primary B cells from peripheral blood | [REMC](http://www.roadmapepigenomics.org/) |
| ChromHMM | chr8:10623800..10649600 | Weak transcription | Blood & T-cell | Primary T helper 17 cells PMA-I stimulated | [REMC](http://www.roadmapepigenomics.org/) |
| ChromHMM | chr8:10623800..10656800 | Weak transcription | Digestive | Gastric | [REMC](http://www.roadmapepigenomics.org/) |
| ChromHMM | chr8:10624000..10649600 | Weak transcription | Other | Pancreas | [REMC](http://www.roadmapepigenomics.org/) |
| ChromHMM | chr8:10624400..10651000 | Quiescent/Low | Other | Placenta Amnion | [REMC](http://www.roadmapepigenomics.org/) |
| ChromHMM | chr8:10624800..10646800 | Weak transcription | Brain | Brain Germinal Matrix | [REMC](http://www.roadmapepigenomics.org/) |
| ChromHMM | chr8:10630800..10662800 | Quiescent/Low | HSC & B-cell | Primary neutrophils fromÃÂ peripheral blood | [REMC](http://www.roadmapepigenomics.org/) |
| ChromHMM | chr8:10634600..10647200 | Weak transcription | Digestive | Fetal Stomach | [REMC](http://www.roadmapepigenomics.org/) |
| ChromHMM | chr8:10635000..10652200 | Weak transcription | Other | Liver | [REMC](http://www.roadmapepigenomics.org/) |
| ChromHMM | chr8:10635400..10664400 | Weak transcription | HSC & B-cell | Primary hematopoietic stem cells | [REMC](http://www.roadmapepigenomics.org/) |
| ChromHMM | chr8:10635600..10656800 | Weak transcription | Other | Ovary | [REMC](http://www.roadmapepigenomics.org/) |
| ChromHMM | chr8:10619200..10656800 | Quiescent/Low | Digestive | Stomach Mucosa | [REMC](http://www.roadmapepigenomics.org/) |
| ChromHMM | chr8:10623600..10667000 | Weak transcription | Thymus | Thymus | [REMC](http://www.roadmapepigenomics.org/) |
| ChromHMM | chr8:10622800..10656000 | Weak transcription | ENCODE | A549 EtOH 0.02pct Lung Carcinoma Cell Line | [REMC](http://www.roadmapepigenomics.org/) |
| ChromHMM | chr8:10630400..10646200 | Weak transcription | ENCODE | GM12878 Lymphoblastoid Cell Line | [REMC](http://www.roadmapepigenomics.org/) |
| ChromHMM | chr8:10621400..10656000 | Weak transcription | ENCODE | HepG2 Hepatocellular Carcinoma Cell Line | [REMC](http://www.roadmapepigenomics.org/) |
| ChromHMM | chr8:10633000..10646400 | Quiescent/Low | ENCODE | NHLF Lung Fibroblast Primary Cells | [REMC](http://www.roadmapepigenomics.org/) |
| ChromHMM | chr8:10634400..10645800 | Weak transcription | ES-deriv | H1 Derived Mesenchymal Stem Cells | [REMC](http://www.roadmapepigenomics.org/) |
| ChromHMM | chr8:10634600..10645800 | Quiescent/Low | IMR90 | IMR90 fetal lung fibroblasts Cell Line | [REMC](http://www.roadmapepigenomics.org/) |
| ChromHMM | chr8:10634800..10645800 | Weak transcription | Mesench | Bone Marrow Derived Cultured Mesenchymal Stem Cells | [REMC](http://www.roadmapepigenomics.org/) |
| ChromHMM | chr8:10635000..10647000 | Weak transcription | ESC | ES-WA7 Cell Line | [REMC](http://www.roadmapepigenomics.org/) |
| ChromHMM | chr8:10635200..10647000 | Quiescent/Low | Sm. Muscle | Colon Smooth Muscle | [REMC](http://www.roadmapepigenomics.org/) |
| ChromHMM | chr8:10635600..10645800 | Quiescent/Low | iPSC | iPS DF 19.11 Cell Line | [REMC](http://www.roadmapepigenomics.org/) |
| ChromHMM | chr8:10635600..10646200 | Weak transcription | Digestive | Esophagus | [REMC](http://www.roadmapepigenomics.org/) |
| ChromHMM | chr8:10636200..10646200 | Quiescent/Low | Brain | Brain Dorsolateral Prefrontal Cortex | [REMC](http://www.roadmapepigenomics.org/) |
| ChromHMM | chr8:10636400..10646200 | Weak transcription | Brain | Brain Inferior Temporal Lobe | [REMC](http://www.roadmapepigenomics.org/) |
| ChromHMM | chr8:10636600..10646200 | Weak transcription | Neurosph | Cortex derived primary cultured neurospheres | [REMC](http://www.roadmapepigenomics.org/) |
| ChromHMM | chr8:10636800..10646200 | Weak transcription | Brain | Brain Hippocampus Middle | [REMC](http://www.roadmapepigenomics.org/) |
| ChromHMM | chr8:10637000..10645800 | Weak transcription | Brain | Brain Angular Gyrus | [REMC](http://www.roadmapepigenomics.org/) |
| ChromHMM | chr8:10637200..10646200 | Weak transcription | Brain | Brain Anterior Caudate | [REMC](http://www.roadmapepigenomics.org/) |
| ChromHMM | chr8:10639600..10646200 | Weak transcription | Neurosph | Ganglion Eminence derived primary cultured neurospheres | [REMC](http://www.roadmapepigenomics.org/) |
| ChromHMM | chr8:10635600..10645800 | Weak transcription | Muscle | Fetal Muscle Trunk | [REMC](http://www.roadmapepigenomics.org/) |
| ChromHMM | chr8:10639800..10645800 | Weak transcription | Muscle | Fetal Muscle Leg | [REMC](http://www.roadmapepigenomics.org/) |
| ChromHMM | chr8:10636200..10646400 | Weak transcription | Other | Placenta | [REMC](http://www.roadmapepigenomics.org/) |
| ChromHMM | chr8:10634400..10646400 | Weak transcription | Other | Lung | [REMC](http://www.roadmapepigenomics.org/) |
| ChromHMM | chr8:10635600..10646600 | Weak transcription | Heart | Right Ventricle | [REMC](http://www.roadmapepigenomics.org/) |
| ChromHMM | chr8:10639800..10646200 | Weak transcription | Muscle | Skeletal Muscle Female | [REMC](http://www.roadmapepigenomics.org/) |
| ChromHMM | chr8:10634400..10646600 | Weak transcription | Other | Spleen | [REMC](http://www.roadmapepigenomics.org/) |
| ChromHMM | chr8:10637200..10646000 | Weak transcription | ENCODE | HSMM cell derived Skeletal Muscle Myotubes Cell Line | [REMC](http://www.roadmapepigenomics.org/) |
| ChromHMM | chr8:10639400..10674000 | Quiescent/Low | Other | Pancreatic Islets | [REMC](http://www.roadmapepigenomics.org/) |
| ChromHMM | chr8:10639800..10648800 | Weak transcription | HSC & B-cell | Primary hematopoietic stem cells short term culture | [REMC](http://www.roadmapepigenomics.org/) |
| ChromHMM | chr8:10639800..10648800 | Weak transcription | HSC & B-cell | Primary hematopoietic stem cells G-CSF-mobilized Female | [REMC](http://www.roadmapepigenomics.org/) |
| ChromHMM | chr8:10639800..10649600 | Weak transcription | HSC & B-cell | Primary hematopoietic stem cells G-CSF-mobilized Male | [REMC](http://www.roadmapepigenomics.org/) |
| ChromHMM | chr8:10639800..10655400 | Quiescent/Low | Blood & T-cell | Primary T CD8+ memory cells from peripheral blood | [REMC](http://www.roadmapepigenomics.org/) |
| ChromHMM | chr8:10640000..10655600 | Quiescent/Low | Other | Fetal Lung | [REMC](http://www.roadmapepigenomics.org/) |
| ChromHMM | chr8:10640200..10669800 | Weak transcription | Heart | Aorta | [REMC](http://www.roadmapepigenomics.org/) |
| ChromHMM | chr8:10640400..10649800 | Weak transcription | Blood & T-cell | Primary T regulatory cells fromÃÂ peripheralÃÂ blood | [REMC](http://www.roadmapepigenomics.org/) |
| ChromHMM | chr8:10641200..10657000 | Weak transcription | Muscle | Psoas Muscle | [REMC](http://www.roadmapepigenomics.org/) |
| ChromHMM | chr8:10641400..10662000 | Weak transcription | Blood & T-cell | Primary T helper naive cells fromÃÂ peripheralÃÂ blood | [REMC](http://www.roadmapepigenomics.org/) |
| ChromHMM | chr8:10641400..10673200 | Quiescent/Low | Brain | Fetal Brain Male | [REMC](http://www.roadmapepigenomics.org/) |
| ChromHMM | chr8:10642000..10650800 | Quiescent/Low | Epithelial | Foreskin Melanocyte Primary Cells skin03 | [REMC](http://www.roadmapepigenomics.org/) |
| ChromHMM | chr8:10642400..10662000 | Quiescent/Low | Blood & T-cell | Primary T helper memory cells from peripheral blood 1 | [REMC](http://www.roadmapepigenomics.org/) |
| ChromHMM | chr8:10643600..10649800 | Weak Repressed PolyComb | Heart | Fetal Heart | [REMC](http://www.roadmapepigenomics.org/) |
| ChromHMM | chr8:10643600..10651200 | Strong transcription | Blood & T-cell | Primary T cells from cord blood | [REMC](http://www.roadmapepigenomics.org/) |
| ChromHMM | chr8:10644200..10661400 | Weak transcription | Epithelial | Foreskin Melanocyte Primary Cells skin01 | [REMC](http://www.roadmapepigenomics.org/) |
| ChromHMM | chr8:10644400..10649400 | Weak transcription | Blood & T-cell | Primary mononuclear cells fromÃÂ peripheralÃÂ blood | [REMC](http://www.roadmapepigenomics.org/) |
| ChromHMM | chr8:10644400..10656800 | Weak transcription | Blood & T-cell | Primary T helper cells PMA-I stimulated | [REMC](http://www.roadmapepigenomics.org/) |
| ChromHMM | chr8:10644200..10653600 | Weak transcription | Heart | Right Atrium | [REMC](http://www.roadmapepigenomics.org/) |
| ChromHMM | chr8:10645000..10670000 | Weak transcription | Digestive | Sigmoid Colon | [REMC](http://www.roadmapepigenomics.org/) |
| ChromHMM | chr8:10644800..10673200 | Quiescent/Low | Digestive | Small Intestine | [REMC](http://www.roadmapepigenomics.org/) |
| ChromHMM | chr8:10639800..10649200 | Weak transcription | Sm. Muscle | Stomach Smooth Muscle | [REMC](http://www.roadmapepigenomics.org/) |
| ChromHMM | chr8:10641200..10665000 | Weak transcription | ENCODE | Dnd41 TCell Leukemia Cell Line | [REMC](http://www.roadmapepigenomics.org/) |
| ChromHMM | chr8:10641600..10650400 | Weak transcription | ENCODE | HeLa-S3 Cervical Carcinoma Cell Line | [REMC](http://www.roadmapepigenomics.org/) |
| ChromHMM | chr8:10640600..10662800 | Weak transcription | ENCODE | Monocytes-CD14+ RO01746 Primary Cells | [REMC](http://www.roadmapepigenomics.org/) |
| ChromHMM | chr8:10641000..10648000 | Enhancers | ES-deriv | hESC Derived CD56+ Mesoderm Cultured Cells | [REMC](http://www.roadmapepigenomics.org/) |
| ChromHMM | chr8:10642200..10646200 | Weak transcription | ES-deriv | H1 BMP4 Derived Mesendoderm Cultured Cells | [REMC](http://www.roadmapepigenomics.org/) |
| ChromHMM | chr8:10642600..10646200 | Weak transcription | ES-deriv | H9 Derived Neuron Cultured Cells | [REMC](http://www.roadmapepigenomics.org/) |
| ChromHMM | chr8:10642800..10646200 | Weak transcription | ES-deriv | H9 Derived Neuronal Progenitor Cultured Cells | [REMC](http://www.roadmapepigenomics.org/) |
| ChromHMM | chr8:10643200..10646600 | Weak transcription | ES-deriv | hESC Derived CD184+ Endoderm Cultured Cells | [REMC](http://www.roadmapepigenomics.org/) |
| ChromHMM | chr8:10643600..10646000 | Weak transcription | ESC | HUES48 Cell Line | [REMC](http://www.roadmapepigenomics.org/) |
| ChromHMM | chr8:10644200..10646200 | Weak transcription | ES-deriv | H1 Derived Neuronal Progenitor Cultured Cells | [REMC](http://www.roadmapepigenomics.org/) |
| ChromHMM | chr8:10644400..10646200 | Weak transcription | ESC | H9 Cell Line | [REMC](http://www.roadmapepigenomics.org/) |
| ChromHMM | chr8:10644600..10646000 | Weak transcription | ES-deriv | hESC Derived CD56+ Ectoderm Cultured Cells | [REMC](http://www.roadmapepigenomics.org/) |
| ChromHMM | chr8:10644600..10646400 | Weak transcription | ESC | H1 Cell Line | [REMC](http://www.roadmapepigenomics.org/) |
| ChromHMM | chr8:10645000..10645800 | Weak transcription | ES-deriv | H1 BMP4 Derived Trophoblast Cultured Cells | [REMC](http://www.roadmapepigenomics.org/) |
| ChromHMM | chr8:10644400..10645800 | Weak transcription | iPSC | iPS-15b Cell Line | [REMC](http://www.roadmapepigenomics.org/) |
| ChromHMM | chr8:10645400..10647800 | Enhancers | iPSC | iPS-20b Cell Line | [REMC](http://www.roadmapepigenomics.org/) |
| ChromHMM | chr8:10644000..10646400 | Weak transcription | iPSC | iPS DF 6.9 Cell Line | [REMC](http://www.roadmapepigenomics.org/) |
| ChromHMM | chr8:10641800..10645800 | Weak transcription | Mesench | Mesenchymal Stem Cell Derived Adipocyte Cultured Cells | [REMC](http://www.roadmapepigenomics.org/) |
| ChromHMM | chr8:10644400..10646200 | Weak transcription | ESC | ES-UCSF4 Cell Line | [REMC](http://www.roadmapepigenomics.org/) |
| ChromHMM | chr8:10642200..10645800 | Weak transcription | Mesench | Adipose Derived Mesenchymal Stem Cell Cultured Cells | [REMC](http://www.roadmapepigenomics.org/) |
| ChromHMM | chr8:10645000..10646600 | Enhancers | Epithelial | Breast Myoepithelial Primary Cells | [REMC](http://www.roadmapepigenomics.org/) |
| ChromHMM | chr8:10644000..10645800 | Weak transcription | Epithelial | Breast variant Human Mammary Epithelial Cells (vHMEC) | [REMC](http://www.roadmapepigenomics.org/) |
| ChromHMM | chr8:10644400..10646200 | Weak transcription | HSC & B-cell | Primary B cells from cord blood | [REMC](http://www.roadmapepigenomics.org/) |
| ChromHMM | chr8:10644800..10647800 | Strong transcription | Blood & T-cell | Primary T cells fromÃÂ peripheralÃÂ blood | [REMC](http://www.roadmapepigenomics.org/) |
| ChromHMM | chr8:10644800..10647600 | Strong transcription | Blood & T-cell | Primary T helper cells fromÃÂ peripheralÃÂ blood | [REMC](http://www.roadmapepigenomics.org/) |
| ChromHMM | chr8:10645000..10647200 | Strong transcription | HSC & B-cell | Primary Natural Killer cells fromÃÂ peripheralÃÂ blood | [REMC](http://www.roadmapepigenomics.org/) |
| ChromHMM | chr8:10642200..10646000 | Weak transcription | Mesench | Mesenchymal Stem Cell Derived Chondrocyte Cultured Cells | [REMC](http://www.roadmapepigenomics.org/) |
| ChromHMM | chr8:10642200..10646000 | Weak transcription | Myosat | Muscle Satellite Cultured Cells | [REMC](http://www.roadmapepigenomics.org/) |
| ChromHMM | chr8:10642400..10647000 | Weak transcription | Other | Fetal Adrenal Gland | [REMC](http://www.roadmapepigenomics.org/) |
| ChromHMM | chr8:10645000..10645800 | Weak transcription | Heart | Left Ventricle | [REMC](http://www.roadmapepigenomics.org/) |
| ChromHMM | chr8:10645200..10646200 | Enhancers | ENCODE | HMEC Mammary Epithelial Primary Cells | [REMC](http://www.roadmapepigenomics.org/) |
| ChromHMM | chr8:10645200..10646200 | Enhancers | ENCODE | HSMM Skeletal Muscle Myoblasts Cell Line | [REMC](http://www.roadmapepigenomics.org/) |
| ChromHMM | chr8:10644800..10646600 | Enhancers | ENCODE | HUVEC Umbilical Vein Endothelial Primary Cells | [REMC](http://www.roadmapepigenomics.org/) |
| ChromHMM | chr8:10645000..10648000 | Genic enhancers | ENCODE | K562 Leukemia Cell Line | [REMC](http://www.roadmapepigenomics.org/) |
| ChromHMM | chr8:10645200..10646400 | Enhancers | ENCODE | NH-A Astrocytes Primary Cells | [REMC](http://www.roadmapepigenomics.org/) |
| ChromHMM | chr8:10642200..10645800 | Weak transcription | ENCODE | NHDF-Ad Adult Dermal Fibroblast Primary Cells | [REMC](http://www.roadmapepigenomics.org/) |
| ChromHMM | chr8:10645600..10645800 | Enhancers | Muscle | Skeletal Muscle Male | [REMC](http://www.roadmapepigenomics.org/) |
| ChromHMM | chr8:10645600..10646000 | Enhancers | Brain | Brain Substantia Nigra | [REMC](http://www.roadmapepigenomics.org/) |
| ChromHMM | chr8:10645600..10646200 | Enhancers | ENCODE | Osteoblast Primary Cells | [REMC](http://www.roadmapepigenomics.org/) |
| ChromHMM | chr8:10645600..10646400 | Enhancers | Epithelial | Foreskin Keratinocyte Primary Cells skin02 | [REMC](http://www.roadmapepigenomics.org/) |
| ChromHMM | chr8:10645600..10646400 | Enhancers | Brain | Brain Cingulate Gyrus | [REMC](http://www.roadmapepigenomics.org/) |
| ChromHMM | chr8:10645600..10647000 | Enhancers | ESC | HUES6 Cell Line | [REMC](http://www.roadmapepigenomics.org/) |
| ChromHMM | chr8:10645600..10647200 | Enhancers | Epithelial | Foreskin Keratinocyte Primary Cells skin03 | [REMC](http://www.roadmapepigenomics.org/) |
| ChromHMM | chr8:10645600..10647400 | Enhancers | iPSC | iPS-18 Cell Line | [REMC](http://www.roadmapepigenomics.org/) |
| ChromHMM | chr8:10645600..10647400 | Enhancers | Adipose | Adipose Nuclei | [REMC](http://www.roadmapepigenomics.org/) |
| ChromHMM | chr8:10645600..10647400 | Enhancers | ENCODE | NHEK-Epidermal Keratinocyte Primary Cells | [REMC](http://www.roadmapepigenomics.org/) |
| ChromHMM | chr8:10645600..10648000 | Enhancers | ESC | ES-I3 Cell Line | [REMC](http://www.roadmapepigenomics.org/) |
| ChromHMM | chr8:10645600..10648000 | Enhancers | ESC | HUES64 Cell Line | [REMC](http://www.roadmapepigenomics.org/) |

Supplementary Table 3. Functional annotations of rs6984094 and variants with r2 >= 0.8 by HaploReg v4.1.

|  | |  | |  | | |  | |  | | |  | |  | |  |  |  |  |  |  |  |
| --- | --- | --- | --- | --- | --- | --- | --- | --- | --- | --- | --- | --- | --- | --- | --- | --- | --- | --- | --- | --- | --- | --- |
| **chr** | **pos (hg38)** | | **LD** | | **LD** | **variant** | | **Ref** | | **SiPhy** | **Promoter** | | **Enhancer** | | **DNAse** | | **Proteins** | **Motifs** | **GRASP QTL** | **Selected eQTL** | **GENCODE** | **dbSNP** |
|  |  |  | **(r²)** | | **(D')** |  |  |  |  | **cons** | **histone marks** | | **histone marks** | |  |  | **bound** | **changed** | **hits** | **hits** | **genes** | **func annot** |
| 8 | 10719265 | | 0.83 | | 0.94 | rs10099100 | | G | |  |  | |  | |  | |  | THAP1 | 1 hit | 13 hits | 4.5kb 3' of SOX7 |  |
| 8 | 10724400 | | 0.83 | | 0.94 | rs58772184 | | C | |  |  | | VAS, SKIN | |  | |  |  |  |  | SOX7 | 3'-UTR |
| 8 | 10724587 | | 0.83 | | 0.94 | rs1139843 | | C | |  |  | | VAS | | SKIN,GI | |  | GATA,Pdx1,RXR::LXR |  |  | SOX7 | 3'-UTR |
| 8 | 10724756 | | 0.83 | | 0.94 | rs1139066 | | C | |  |  | | VAS | |  | |  | 5 altered motifs |  |  | SOX7 | 3'-UTR |
| 8 | 10727704 | | 0.83 | | 0.94 | rs11784335 | | T | |  | 4 tissues | | 6 tissues | |  | |  | 4 altered motifs |  |  | SOX7 | intronic |
| 8 | 10734547 | | 0.83 | | 0.94 | rs10110723 | | C | |  |  | |  | | 20 tissues | | CTCF,RAD21 | Rad21 |  |  | SOX7 |  |
| 8 | 10740020 | | 0.9 | | 1 | rs140159729 | | A | |  |  | |  | |  | | SETDB1 | Gfi1,Mef2,TATA |  |  | SOX7 |  |
| 8 | 10741194 | | 0.9 | | 1 | rs2163381 | | T | |  |  | |  | |  | |  | 11 altered motifs |  |  | SOX7 |  |
| 8 | 10744613 | | 0.95 | | 1 | rs59275830 | | C | |  |  | |  | |  | |  | Pax-4 |  |  | SOX7 |  |
| 8 | 10745889 | | 0.95 | | 1 | rs6601515 | | T | |  |  | |  | |  | |  | 18 altered motifs |  |  | SOX7 |  |
| 8 | 10746460 | | 0.95 | | 1 | rs73540634 | | A | |  |  | |  | |  | |  | 11 altered motifs |  |  | SOX7 |  |
| 8 | 10748360 | | 0.95 | | 1 | rs28401657 | | C | |  |  | |  | |  | |  | 5 altered motifs |  |  | SOX7 |  |
| 8 | 10749291 | | 0.95 | | 1 | rs4841439 | | T | |  |  | |  | |  | |  | 11 altered motifs |  |  | SOX7 |  |
| 8 | 10750849 | | 0.95 | | 1 | rs58297791 | | C | |  |  | |  | |  | |  | Dobox4,Hdx,Irf |  |  | SOX7 |  |
| 8 | 10752202 | | 0.95 | | 1 | rs13250710 | | T | |  |  | |  | |  | |  | 15 altered motifs |  |  | SOX7 |  |
| 8 | 10755415 | | 0.9 | | 1 | rs4840514 | | G | |  |  | |  | |  | |  | Ets,NRSF,Znf143 |  | 7 hits | SOX7 |  |
| 8 | 10756558 | | 0.9 | | 1 | rs73542533 | | G | |  |  | | 4 tissues | | ESDR | |  | 23 altered motifs |  |  | SOX7 |  |
| 8 | 10760690 | | 0.9 | | 1 | rs4841440 | | T | |  | 4 tissues | | 8 tissues | | BRST | |  | EWSR1-FLI1,STAT |  |  | SOX7 |  |
| 8 | 10763551 | | 0.85 | | 1 | rs2898250 | | G | |  |  | | 4 tissues | | BRST | |  | 5 altered motifs |  |  | SOX7 |  |
| 8 | 10765628 | | 0.9 | | 1 | rs1078543 | | T | |  |  | |  | | PLCNT | |  | RXRA,SP1 |  |  | PINX1 | missense |
| 8 | 10766374 | | 0.9 | | 1 | rs891560 | | T | |  |  | | 4 tissues | | GI | |  | NRSF,Sin3Ak-20,Zbtb12 |  |  | PINX1 | intronic |
| 8 | 10767489 | | 0.95 | | 1 | rs7350075 | | C | |  |  | | ESC, ESDR, BRST | |  | |  | HNF1,Irf |  | 9 hits | PINX1 | intronic |
| 8 | 10767535 | | 0.9 | | 1 | rs7350124 | | T | |  |  | | ESDR | |  | |  | SREBP |  | 9 hits | PINX1 | intronic |
| 8 | 10767670 | | 0.9 | | 1 | rs73544348 | | T | |  |  | | ESDR | | IPSC,ADRL | |  | LBP-1,NF-Y |  |  | PINX1 | intronic |
| 8 | 10767697 | | 0.9 | | 1 | rs75664050 | | T | |  |  | | ESDR | |  | |  | AP-1,STAT |  |  | PINX1 | intronic |
| 8 | 10768823 | | 0.95 | | 1 | rs17774023 | | T | |  |  | | ESDR | |  | |  |  |  | 9 hits | PINX1 | intronic |
| 8 | 10770640 | | 0.95 | | 1 | rs10503412 | | G | |  |  | |  | |  | |  | SIX5,Zfp187,Znf143 |  |  | PINX1 | intronic |
| 8 | 10772368 | | 0.95 | | 1 | rs4240668 | | C | |  |  | | BLD, ADRL, LNG | | 5 tissues | |  | Myb | 1 hit |  | PINX1 | intronic |
| 8 | 10773158 | | 0.95 | | 1 | rs17152366 | | T | |  |  | | BLD, SKIN | |  | |  | Sox | 1 hit |  | PINX1 | intronic |
| 8 | 10779458 | | 1 | | 1 | rs34247687 | | CATA | |  |  | | 6 tissues | |  | |  | 4 altered motifs |  |  | PINX1 | intronic |
| 8 | 10779642 | | 1 | | 1 | rs7836178 | | T | |  |  | | 6 tissues | |  | |  | Cdc5,Mef2 |  |  | PINX1 | intronic |
| 8 | 10780846 | | 1 | | 1 | rs7008259 | | A | |  |  | |  | |  | |  | 12 altered motifs |  |  | PINX1 | intronic |
| 8 | 10782785 | | 1 | | 1 | rs35055625 | | AT | |  |  | | VAS, BLD | | ESDR | | GATA2 | 7 altered motifs |  |  | PINX1 | intronic |
| 8 | 10783969 | | 1 | | 1 | rs10096939 | | C | |  |  | |  | | 11 tissues | | 4 bound proteins | 14 altered motifs |  |  | PINX1 | intronic |
| 8 | 10785588 | | 1 | | 1 | rs2409664 | | C | |  |  | | 4 tissues | |  | |  | AP-2rep,Ik-2 |  |  | PINX1 | intronic |
| 8 | 10787223 | | 1 | | 1 | rs11778578 | | A | |  |  | | ESDR, HRT | |  | |  | 5 altered motifs |  |  | PINX1 | intronic |
| 8 | 10787604 | | 1 | | 1 | rs17152410 | | T | |  |  | | 4 tissues | | BRN | |  |  |  |  | PINX1 | intronic |
| 8 | 10788228 | | 1 | | 1 | rs6984094 | | T | |  |  | | 11 tissues | | VAS | |  | TATA |  |  | PINX1 | intronic |
| 8 | 10789524 | | 1 | | 1 | rs11783803 | | G | |  | MUS | | 19 tissues | | 15 tissues | |  | 4 altered motifs |  |  | PINX1 | intronic |
| 8 | 10790083 | | 1 | | 1 | rs17152412 | | C | |  | MUS | | 16 tissues | | 5 tissues | |  | Myf,p300 |  |  | PINX1 | intronic |
| 8 | 10790576 | | 1 | | 1 | rs73546269 | | A | |  | BRN | | 9 tissues | | ESDR,ESC | | CTCF,RAD21 | ERalpha-a,Egr-1,p300 |  |  | PINX1 | intronic |
| 8 | 10791685 | | 1 | | 1 | rs11779090 | | C | |  |  | |  | |  | |  | 4 altered motifs |  |  | PINX1 | intronic |
| 8 | 10792189 | | 1 | | 1 | rs10481452 | | C | |  |  | |  | |  | |  | ERalpha-a,GR,HNF4 |  |  | PINX1 | intronic |
| 8 | 10794396 | | 0.89 | | 0.94 | rs10503411 | | G | |  |  | |  | |  | |  | AIRE,En-1 |  |  | PINX1 | intronic |
| 8 | 10794617 | | 0.89 | | 0.94 | rs2409660 | | G | |  |  | | 4 tissues | |  | | CTCF | Ets,ZBTB33 |  |  | PINX1 | intronic |
| 8 | 10795112 | | 0.89 | | 0.94 | rs4840515 | | T | |  |  | | 4 tissues | |  | |  | HP1-site-factor,Pou2f2,TEF |  |  | PINX1 | intronic |
| 8 | 10797820 | | 0.89 | | 0.94 | rs6983956 | | G | |  |  | | ESDR, PLCNT, VAS | | KID | |  | 8 altered motifs | 1 hit |  | PINX1 | intronic |
| 8 | 10799415 | | 0.89 | | 0.94 | rs10089548 | | C | |  |  | |  | | 10 tissues | |  | E2A,RP58 |  |  | PINX1 | intronic |

Supplementary table 4. The associations between genotypes of rs6984094 and rs7726159, and clinical manifestations of SLE patients in the replication cohort.

| Clinical parameters | rs6984094 | | |  | rs7726159 | | | |
| --- | --- | --- | --- | --- | --- | --- | --- | --- |
|  | CT (n=19) | TT (n=948) | *p*-value |  | AA (n=212) | CA (n=508) | CC (n=265) | *p*-value |
| Age onset (mean±SD) | 31.7±13.8 | 31.3±13.0 | 0.90 |  | 32.0±12.9 | 31.1±12.9 | 31.1±12.9 | 0.67 |
| Sex (female, %) | 18 (94.7) | 883 (93.1) | 1.00^a^ |  | 191 (90.1) | 474 (93.3) | 265 (94.3) | 1.00^a^ |
| Malar rash (+, %) | 5 (26.3) | 236 (24.9) | 1.00^a^ |  | 53 (25.0) | 132 (26.0) | 68 (24.2) | 1.00^a^ |
| Discoid rash (+, %) | 0 (0) | 7 (0.7) | 1.00^a^ |  | 2 (0.9) | 5 (1.0) | 0 (0) | 1.00^a^ |
| Photosensitivity (+, %) | 2 (10.5) | 39 (4.1) | 0.17^a^ |  | 6 (2.8) | 30 (5.9) | 6 (2.1) | 0.43^a^ |
| Oral ulcers (+, %) | 1 (5.3) | 71 (7.5) | 1.00^a^ |  | 18 (8.5) | 35 (6.9) | 20 (7.1) | 1.00^a^ |
| Arthritis (+, %) | 10 (52.6) | 256 (27.0) | 0.03^a^ |  | 65 (30.7) | 143 (28.1) | 71 (25.3) | 0.02 |
| Serositis (+, %) | 1 (5.3) | 80 (8.4) | 1.00^a^ |  | 20 (9.4) | 43 (8.5) | 20 (7.1) | 0.62^a^ |
| Renal disorder (+, %) | 9 (47.4) | 487 (51.5) | 0.90 |  | 108 (51.4) | 258 (50.8) | 142 (50.7) | 0.72 |
| Neurological disorder (+, %) | 0 (0) | 35 (3.7) | 0.82^a^ |  | 11 (5.2) | 19 (3.7) | 6 (2.1) | 0.82 |
| Hematological disorder (+, %) | 8 (50.0) | 510 (55.1) | 0.88 |  | 107 (52.2) | 287 (57.7) | 149 (54.6) | 0.68 |
| Anti-dsDNA antibodies (+, %) | 11 (64.7) | 544 (63.6) | 0.93 |  | 122 (63.5) | 306 (66.1) | 142 (57.0) | 0.92 |
| SLEDAI (mean±SD) | 4.5±4.6 | 4.7±4.1 | 0.96 |  | 4.7±4.2 | 4.8±4.0 | 4.3±4.1 | 0.24 |

a: P-value calculated after Continuity Correction for 1 cell (25%) have expected count less than 5.
